# Femoral resection accuracy and precision in manual caliper‐verified kinematic alignment total knee arthroplasty

**DOI:** 10.1002/jeo2.70234

**Published:** 2025-04-17

**Authors:** David Forrest Scott, Emma N. Horton

**Affiliations:** ^1^ Spokane Joint Replacement Center, Inc. Spokane Washington USA; ^2^ Washington State University Elson S. Floyd College of Medicine Spokane Washington USA

**Keywords:** kinematic alignment, patient‐specific instrumentation, robotic arm‐assisted, total knee arthroplasty

## Abstract

**Introduction:**

The accuracy and precision of bone resections in total knee arthroplasty (TKA) are essential to avoid poor implant positioning, which can lead to component wear, pain, and instability, reducing patient satisfaction and implant survivorship. Technology‐assisted TKA techniques aim to improve accuracy but come with added costs, increased operative time, and varying success in clinical outcomes. Caliper‐verified kinematic alignment (KA) attempts to restore the joint line by precisely measuring resections to equal implant thickness. We evaluated the accuracy and precision of caliper‐verified KA‐TKA performed with manual instruments. We hypothesised that this technique would achieve high accuracy and precision, with an average absolute difference between actual and target distal and posterior femoral resection measurements of ≤ 0.5 mm.

**Methods:**

385 consecutive patients underwent primary unrestricted caliper‐verified KA‐TKA with manual instrumentation. The thickness of the distal medial (DM), distal lateral (DL), posterior medial (PM) and posterior lateral (PL) femoral condyle resections were measured with a caliper and compared to a target determined by the degree of cartilage loss, saw blade kerf, and femoral component thickness.

**Results:**

The mean differences between the resected and target thicknesses for DM, DL, PM and PL femoral resections were 0.1 ± 0.2 mm, 0.1 ± 0.3 mm, 0.3 ± 0.5 mm and 0.2 ± 0.4 mm, respectively (mean ± std. dev.). Most femoral resections were within 0.5 mm of the target—97.7%, 94.5%, 85.7% and 89.4% of DM, DL, PM and PL resections, respectively.

**Conclusion:**

Manual caliper‐verified KA‐TKA achieved highly accurate and precise femoral resections with absolute differences from target that averaged 0.175 mm. This simple, logical, efficient, and reproducible surgical technique may be an option for surgeons contemplating the use of technology‐assisted options, such as patient‐specific instrumentation or robotic arm‐assisted TKA, and surgeons without access to such technologies.

**Level of Evidence:**

Level II.

**Abbreviations**
BMI: body mass index
CT: computed tomography
DL: distal lateral
DM: distal medial
KA: kinematic alignment
MA: mechanical alignment
MRI: magnetic resonance imaging
PL: posterior lateral
PM: posterior medial
PSI: patient‐specific instruction
RAA: robotic arm‐assisted
SD: standard deviation
TKA: total knee arthroplasty

## INTRODUCTION

Accurate bone resections are essential for proper component alignment and optimal outcomes in total knee arthroplasty (TKA). Poor implant alignment can lead to increased wear, loosening, pain and instability, reducing patient satisfaction and implant survivorship [1, 2, 3]. Technology‐assisted TKA techniques were developed to enhance reproducibility and accuracy by limiting human error and reducing outliers. However, their benefits are debatable [1, 2, 3, 4, 5, 6, 7, 8, 9, 10, 11, 12, 13, 14, 15, 16, 17, 18, 19], as many variables impact their accuracy and precision [8, 20, 21, 22, 23, 24, 25, 26, 27]. Manual instrumentation paired with caliper verification for kinematic alignment (KA) remains a viable and widely used alternative [8, 28, 29, 30, 31, 32].

Caliper‐verified KA aims to restore each patient's pre‐arthritic joint line without soft tissue releases, by removing the same composite thickness of cartilage, bone and saw blade kerf as the implant. The femur defines the kinematic axes of the knee, making the accuracy and precision of the femoral resections critical to the procedure's technical outcome. Mechanical alignment (MA), the primary alternative to KA, applies a uniform alignment target to all patients, disregarding individual joint line variability and resection thickness.

KA‐TKA can be performed with manual instrumentation or technology assistance, such as patient‐specific instrumentation (PSI‐TKA) and robotic arm‐assisted (RAA‐TKA). PSI‐TKA involves customised cutting blocks based on three‐dimensional patient models [4, 5], while RAA‐TKA employs robotic systems to assist in bone resections within predefined limits [5]. These methods often rely on computed tomography (CT) or magnetic resonance imaging (MRI) to generate three‐dimensional models [4]. CT imaging exposes patients to additional radiation [6].

Compared to manual TKA, RAA‐TKA and PSI‐TKA can increase surgery team anxiety, operative time, and costs [16, 17, 33, 34, 35, 36, 37, 38, 39]. Christen et al. [34] estimated that PSI‐TKA decreases operative time by five minutes, but increase costs by $1520 due to the disposable guides and preoperative imaging. Image‐based and imageless RAA‐TKA increase the operating time by 14 and 25 min, and costs by $2600 and $1530, respectively [34]. Over 5 years, robot technology expenses may reach USD 1.362 million due to imaging, disposables, and technical support [37], substantially limiting access across different markets and countries [8]. It is unclear whether the cost of these technologies are justified by improvements in outcomes [16, 17, 34, 35, 36, 38, 39].

Reports of the accuracy of TKA technology‐assistance are mixed. Some studies indicate that RAA‐TKA improves implant positioning without enhancing clinical results [40, 41, 42, 43, 44, 45, 46], while PSI‐TKA has shown no improvement [47, 48, 49] or even inferior accuracy [1, 2, 3, 4, 5, 8, 12, 13, 18]. Most studies reporting TKA accuracy and precision focus on angular bone cuts to attain a neutral mechanical axis—the standard of MA. Few studies evaluate the accuracy and precision of resection thickness, making comparisons between manual and technology‐assisted approaches difficult.

To establish a baseline, we evaluated the accuracy and precision of caliper‐verified KA using manual instrumentation by measuring the mean differences between target and actual femoral resections. We hypothesised that manual KA‐TKA would achieve high accuracy and precision, with an average absolute difference measuring ≤ 0.5 mm.

## METHODS

The study cohort consisted of all primary TKA patients who consented to participation in a registry database between April 2020 and April 2024. The study enroled 385 consecutive patients, including 176 women and 209 men. The cohort's mean age and body mass index (BMI) at surgery were 67 years (41–89, standard deviation [SD] 8.59) and 32.4 kg/m^2^ (range 21.46–48.40, SD 4.95) respectively. The average preoperative hip‐knee‐ankle angle was 175° (range 163°–196°, SD 6.29°. The average tourniquet time was 39.2 minutes (range 21–64, SD 7.06).

Each consenting participant underwent primary TKA with unrestricted KA by a single high‐volume surgeon who has performed over 5000 manually instrumented KA‐TKA surgeries; no patients were excluded. The following manually instrumented surgical technique has been described previously [28, 30, 32] and was utilised in all cases: tourniquet inflation to 300 mmHg before skin incision, a medial parapatellar arthrotomy, caliper‐verified measured femoral resection with posterior referencing, manual cutting blocks with removable saw captures for accurate pre‐resection visual assessment, a 1.40 mm saw capture slot with a length of 14.8 mm, Stryker Precision Falcon oscillating‐tip saw blades with a thickness of 1.27 mm and cut edge 19.5 mm wide (Stryker Instruments, Portage, MI, USA), cement fixation, and Medacta GMK Sphere (Medacta, Castel San Pietro, Switzerland) implants.

To recreate each patient's native joint line obliquity and knee kinematics, the femur was positioned in neutral rotation to the posterior condyles. The target resection thickness was defined as equal to the thickness of the prosthetic femoral condyle minus one millimetre for bone removal due to the kerf of the saw blade. Adjustments were made for cartilage loss (worn or unworn), assuming a 2 mm normal cartilage thickness. The thicknesses of the distal and posterior condyles of the specific TKA device used in this study were 9 mm and 8 mm, respectively. Therefore, the target for the distal resection was 8 mm for unworn cartilage and 6 mm for worn cartilage, while the target for the posterior resection was 7 mm for unworn and 5 mm for worn. If there was partial thickness cartilage loss, a ring curette was utilised to scrape the remaining cartilage down to bone. The anatomic position of the individual patient's joint line was matched by using a 2 mm shim on any worn condyle. To properly position the 4/1 cutting block, the shim was added to the drill guide to place the distal cut block and to the posterior runners of the anteroposterior sizer for the posterior femoral condyles. No restrictions were implemented based on preoperative alignment.

**Figure 1:**
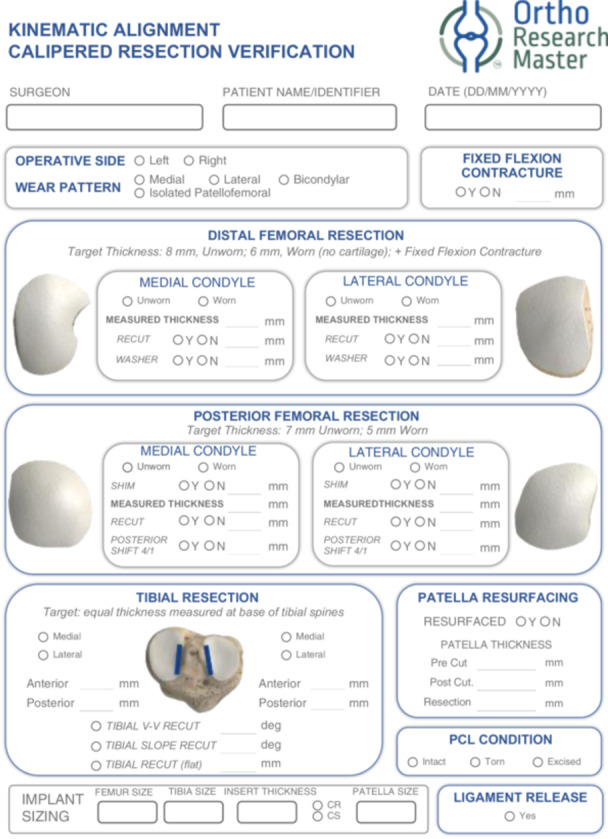
Kinematic alignment calipered resection verification form.

The following sequence of resections was performed in every case: distal femoral, posterior femoral, anterior and chamfer resections. The distal and posterior resections were measured immediately after the respective cuts were made to allow any necessary corrections before the subsequent resection. The thickness of the distal medial (DM), distal lateral (DL), posterior medial (PM) and posterior lateral (PL) femoral condyle resections were measured with a standardised manual vernier caliper with a resolution of one‐half millimetre following a previously established and accepted published technique [20, 50, 51, 52, 53]. Recent findings from a controlled and blinded trial indicate that this caliper measurement technique achieves a precision of 0.2 mm, better than the caliper's resolution of 0.5 mm. The technique also shows a negligible bias or systematic error, along with excellent intra‐class correlation coefficients for repeatability and reproducibility, exceeding 0.95 (AJ Nedopil, personal communication, 16 February 2025). The difference between the target thickness and actual measured resection was then calculated. A sign was used to distinguish between under‐ and over‐resection, but the *absolute* values were utilised for all mathematical evaluations. Data was captured in a resection verification form (Figure 1) and analysed using Ortho Research Master™ electronic data capture and analysis software (Spokane Joint Replacement Center, Inc., Spokane, WA, USA).

Any actual resection measurement differing from the target by ≥ 1.0 mm was adjusted with either a recut, washer, or block shift according to the following protocol. Undercut distal and posterior femoral condyle resections were recut. Overcut distal condyles were treated with the placement of a washer under the 4/1 cut block, while overcut posterior condyles were treated with a posterior shift of the 4/1 cut block. Cut block shifts were made in one‐millimetre increments. For example, if the target posterior condyle resection was 7 mm, and 8 mm was measured, a hole redrilling guide would be utilised to drill new cut block pin locating holes to shift the 4/1 block 1 mm posteriorly. The 4/1 block is then impacted into these two new holes, moving its position one mm more posterior. The subsequent cuts would shift the placement of the femoral component 1 mm posteriorly and leave a 1 mm gap at the interface between the posterior condyle bone and implant to be filled with cement.

### Ethics

The WCG Institutional Review Board approved this knee registry study. All eligible participants provided written informed consent and good clinical practice regulatory mandates were followed.

## RESULTS

Most manual caliper‐verified KA‐TKA resections were within ±0.5 mm of the target—97.7%, 94.5%, 85.7% and 89.4% of DM, DL, PM and PL femoral resections, respectively. Moreover, 72.9% of resections were on target, including 82.3%, 76.6%, 64.2% and 68.1% of DM, DL, PM and PL femoral resections, respectively (Table 1).

**Table 1:** Manual kinematic alignment overcut and undercut femoral resections (*N* = 385 subjects).

Femoral resection	On target	Overcut	Undercut
Distal medial (DM)	319 (82.9%)	44 (11.4%)	22 (5.7%)
Distal lateral (DL)	295 (76.6%)	16 (4.2%)	74 (19.2%)
Posterior medial (PM)	247 (64.2%)	50 (13.0%)	88 (22.9%)
Posterior lateral (PL)	262 (68.1%)	43 (11.2%)	80 (20.8%)
Average	(72.9%)	(9.9%)	(17.1%)

**Figure 2:**
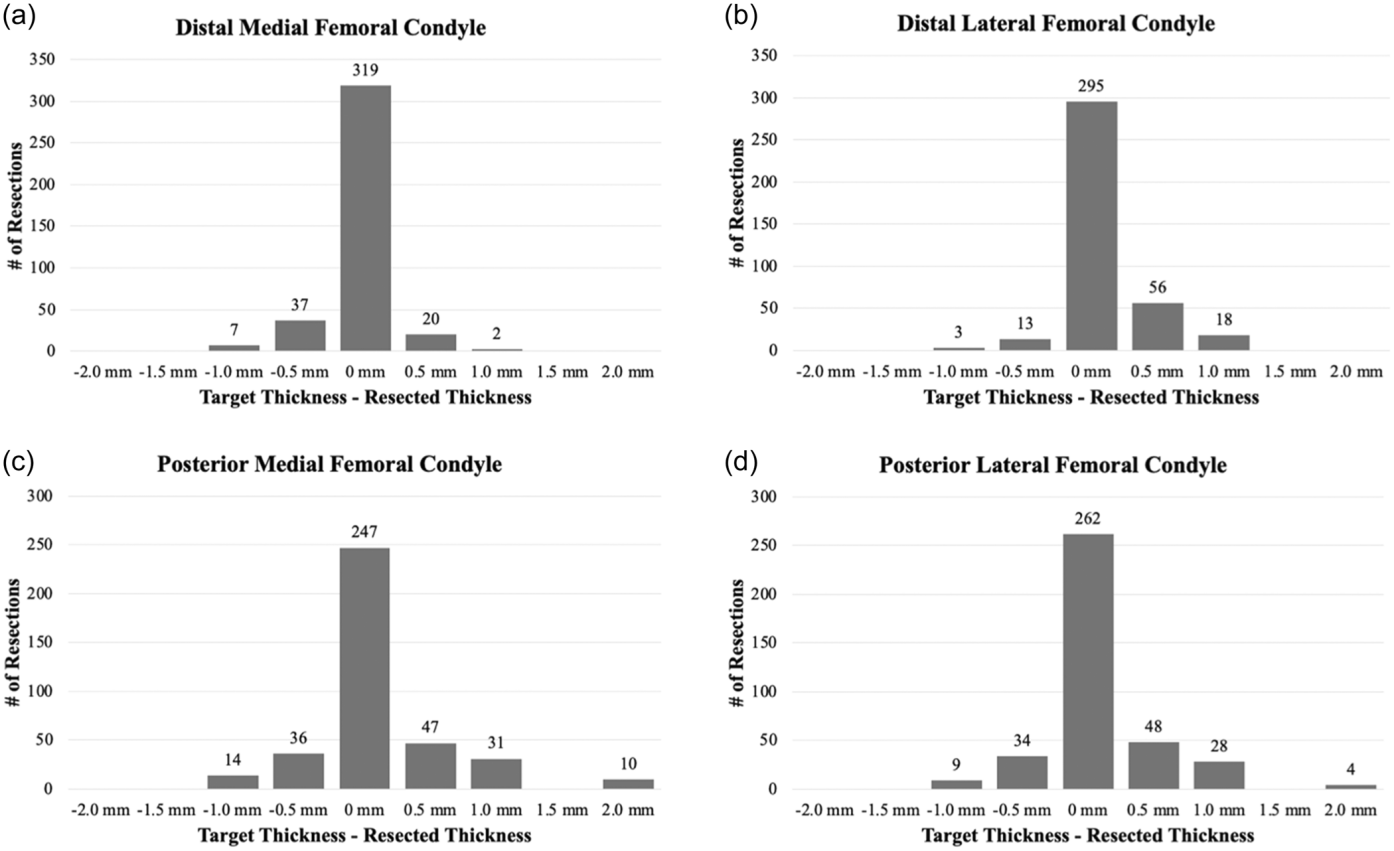
Target and actual resection difference distributions by femoral condyle position.

9.9% of the remaining resections were overcut (exceeded target thickness), and 17.1% were undercut (less than target thickness). The mean *signed* differences (under‐ vs over‐cut) between the target and actual resected thicknesses for the manual KA DM, DL, PM and PL femoral resections were −0.04 ± 0.2 mm (overcut), 0.1 ± 0.3 mm (undercut), 0.1 ± 0.5 mm (undercut), and 0.1 ± 0.4 mm (undercut), respectively (Table 2). The differences ranged from −1 mm to 1 mm for the DM and DL resections and −1 mm to 2 mm for the PM and PL resections; frequency histograms are displayed in Figure 2.

**Table 2:** Actual mean *signed* accuracy of manual caliper‐verified kinematic alignment total knee arthroplasty (TKA) (*N* = 385 subjects).

Femoral resection position	Mean ± SD (mm)	Range (mm)
Distal medial (DM)	−0.04 ± 0.2	−1 (min.), 1 (max)
Distal lateral (DL)	0.1 ± 0.3	−1 (min.), 1 (max)
Posterior medial (PM)	0.1 ± 0.5	−1 (min.), 2 (max)
Posterior lateral (PL)	0.1 ± 0.4	−1 (min.), 2 (max)

The mean *absolute* differences between the resected and target thicknesses for the DM, DL, PM and PL femoral resections were 0.1 ± 0.2 mm, 0.1 ± 0.3 mm, 0.3 ± 0.5 mm and 0.2 ± 0.4 mm, respectively (mean ± SD) (Table 3), with an overall average of 0.175 mm ± 0.350.

**Table 3:** Mean *absolute* accuracy of manual caliper‐verified kinematic alignment total knee arthroplasty (TKA) (*N* = 385 subjects).

Femoral resection	Absolute mean ± SD (mm)	Within 0.5 mm	Within 1.0 mm	Within 2.0 mm
Distal medial (DM)	0.1 ± 0.2	97.7%	100%	100%
Distal lateral (DL)	0.1 ± 0.3	94.5%	100%	100%
Posterior medial (PM)	0.3 ± 0.5	85.7%	97.4%	100%
Posterior lateral (PL)	0.2 ± 0.4	89.4%	99.1%	100%

Overcut and undercut resections were selectively corrected with recuts, washers, or posterior shifts (Table 4). Recuts were performed for 6.8%, 10.9%, 2.1% and 1.8% of undercut DM, DL, PM and PL resections, respectively. 89.2% of all recuts were 1 mm or less. One (0.3%) one‐millimetre washer was utilised for an overcut DL resection. Posterior shifts were used for 0.5% and 0.8% of PM and PL resections, respectively.

**Table 4:** Use of recuts, washers and posterior shifts to address overcut and undercut resections.

Resection position	Distal medial	Distal lateral	Posterior medial	Posterior lateral
Recut	26 (6.8%)	42 (10.9%)	8 (2.1%)	7 (1.8%)
Mean recut thickness (mm)	1.0	1.0	1.1	1.3
Washer	0 (0.0%)	1 (0.3%)	0 (0.0%)	0 (0.0%)
Mean washer thickness (mm)	‐	1.0	‐	‐
Posterior shift	‐	‐	2 (0.5%)	3 (0.8%)
Mean posterior shift (mm)	‐	‐	2.0	1.5

## DISCUSSION

Our findings support the primary hypothesis that caliper‐verified KA‐TKA performed with manual instruments produces excellent femoral resection accuracy and precision, with the absolute difference between target and actual resection measurements averaging 0.175 ± 0.350 mm. We also found a high rate of initial on‐target resections of 73%, a total recut rate (all four individual resections summed) of 22%, and a very low washer/posterior shift rate of 1.6%. Our data is consistent with existing manual KA‐TKA literature [52, 54], and demonstrates that this technique may be as accurate and precise as any currently available options, including PSI‐ and RAA‐TKA [55, 56, 57, 58, 59, 60, 61, 62, 63, 64, 65, 66, 67, 68, 69, 70, 71]. The present report of a large dataset from a single high‐volume surgeon adds to the literature additional novel unpublished information.

We performed a literature search for all comparative femoral resection data from technology‐assisted TKA studies reporting absolute mean differences between target and actual resections. We identified thirteen RAA‐TKA papers [55, 56, 57, 58, 60, 61, 62, 63, 65, 66, 69, 70, 71] and four PSI‐TKA papers [59, 64, 67, 68]‐including one with two cohorts [67], reporting femoral resection accuracy and precision. Of the 13 RAA‐TKA papers, five were cadaver studies [56, 57, 61, 63, 70], and eight were clinical [55, 58, 60, 62, 65, 66, 69, 71]. Six studies (three cadaver studies [56, 57, 63] and three clinical studies [58, 62, 66]) were excluded for allowing over‐ versus under‐cuts to cancel each other arithmetically, inappropriately under‐reporting mean absolute differences and standard deviations. Among the 13 RAA‐TKA studies, two used the MAKO system (one rejected), five used ROSA (two rejected) and six used other systems (two rejected).

Our mean and SD difference from target averaged for all four femoral cuts was 0.175 ± 0.350, respectively, that of the accepted RAA‐TKA literature 0.754 ± 0.697, and that of the PSI literature 1.133 ± 1.171, respectively (Tables 5, 6, 7). This literature review revealed substantial methodological heterogeneity, a lack of comparative cohorts, and compromised statistical methods, yet all concluded that PSI‐ or RAA‐TKA femoral resections were highly accurate. These shortcomings in the femoral resection accuracy and precision literature limit our analysis and call for better uniformity and improved scientific rigour in reporting future results. Nonetheless, acknowledging the limitations of this thorough comparative literature review, there are no reports of femoral resection accuracy or precision that equal or exceed the data in the present study.

**Table 5:** Absolute mean accuracy and precision comparison of femoral condyle resections for RAA‐TKA compared to KA‐TKA with manual instrumentation Welch's *t*‐test determined whether the mean accuracy significantly differed, and an F‐test determined whether the standard deviations (SD) of RAA significantly differed from manual KA (bold* indicates *p* ≤ 0.05, not significant denoted by *italics*).

RAA‐TKA study ID	*N*	Distal medial accuracy ± SD (mm)	Distal lateral accuracy ± SD (mm)	Posterior medial accuracy ± SD (mm)	Posterior lateral accuracy ± SD (mm)
**In vivo studies**					
**KA‐manual instrumentation**	**385**	**0.1** ± **0.2 (ref.)**	**0.1** ± **0.3 (ref.)**	**0.3** ± **0.5 (ref.)**	**0.2** ± **0.4 (ref.)**
Rossi et al. [1]	75	**0.8** ^*^ ± **0.6** ^*^	**0.9** ^*^ ± **0.7** ^*^	*0.4* ± **0.6** ^*^	**0.6** ^*^ ± **0.5** ^*^
Xia et al. [2]	31	**0.9** ^*^ ± **0.7** ^*^	**1.0** ^*^ ± **0.7** ^*^	**0.7** ^*^ ± *0.5*	**1.0** ^*^ ± **0.8** ^*^
Wan et al. [3]	28	**1.3** ^*^ ± **0.8** ^*^	**1.3** ^*^ ± **0.9** ^*^	**0.8** ^*^ ± *0.6*	**0.9** ^*^ ± **0.7** ^*^
Li et al. [4]	36	**0.4** ^*^ ± **0.6** ^*^	**0.5** ^*^ ± **0.7** ^*^	**0.6** ^*^ ± **0.8** ^*^	**0.7** ^*^ ± **0.8** ^*^
Gamie et al. [5]	44	0.7* ± 0.6*	0.9* ± 1.2*	1.1* ± 1.0*	1.0* ± 0.8*
**Cadaveric studies**					
**KA‐manual instrumentation**	**385**	**0.1** ± **0.2 (ref.)**	**0.1** ± **0.3 (ref.)**	**0.3** ± **0.5 (ref.)**	**0.2** ± **0.4 (ref.)**
Li et al. [6]	10	**0.4** ^*^ ± *0.2*	**0.4** ^*^ ± *0.2*	*0.3* ± *0.3*	*0.3* ± *0.3*
Seidenstein et al. [7]	15	**0.7** ^*^ ± **0.7** ^*^	**0.7** ^*^ ± **0.7** ^*^	**0.6** ^*^ ± *0.5*	**0.6** ^*^ ± *0.5*
Xia et al. [8]	20	**0.6** ^*^ ± **0.5** ^*^	**0.6** ^*^ ± **0.4** ^*^	**0.6** ^*^ ± *0.4*	**0.8** ^*^ ± **0.7** ^*^

Abbreviations: KA, kinematic alignment; RAA, robotic arm‐assisted; TKA, total knee arthroplasty.

**Table 6:** Absolute mean accuracy and precision comparison of femoral condyle resections for PSI‐TKA compared to KA‐TKA with manual instrumentation Welch's *t*‐test determined whether the mean accuracy significantly differed, and an F‐test determined whether the standard deviations (SD) of PSI significantly differed from manual KA (bold* indicates *p* ≤ 0.05, not significant denoted by *italics*).

PSI‐TKA study ID	*N*	Distal medial accuracy ± SD (mm)	Distal lateral accuracy ± SD (mm)	Posterior medial accuracy ± SD (mm)	Posterior lateral accuracy ± SD (mm)
**KA‐TKA manual instrumentation**	**385**	**0.1** ± **0.2 (ref.)**	**0.1** ± **0.3 (ref.)**	**0.3** ± **0.5 (ref.)**	**0.2** ± **0.4 (ref.)**
Wernecke et al. [68]	118	**0.9** ^*^ ± **1.3** ^*^	**0.9** ^*^ ± **1.3** ^*^	**1.5** ^*^ ± **2.1** ^*^	**0.8** ^*^ ± **1.2** ^*^
Kim et al. [59]	30	**0.8** ^*^ ± **0.4** ^*^	**0.7** ^*^ ± **0.4** ^*^	**0.8** ^*^ ± *0.5*	**0.8** ^*^ ± *0.5*
Kang et al. (MRI) [67]	36	**0.9** ^*^ ± **0.7** ^*^	**1.4** ^*^ ± **0.9** ^*^	**1.1** ^*^ ± **0.9** ^*^	**1.3** ^*^ ± **0.9** ^*^
Kang et al. (CT) [67]	35	**1.2** ^*^ ± **0.7** ^*^	**1.6** ^*^ ± **1.0** ^*^	**1.4** ^*^ ± **0.9** ^*^	**1.6** ^*^ ± **0.8** ^*^
Yamamura et al. [64]	45	**0.6** ^*^ ± **0.7** ^*^	**1.4** ^*^ ± **1.2** ^*^	**1.8** ^*^ ± **0.9** ^*^	**1.7** ^*^ ± **1.0** ^*^

Abbreviations: KA, kinematic alignment; PSI, patient‐specific instrumentation; TKA, total knee arthroplasty.

**Table 7:** Absolute mean accuracy comparison of four femoral condyle resections combined for KA‐TKA with manual instrumentation compared to RAA‐TKA cadaver studies, RAA‐TKA clinical studies, RAA‐TKA studies combined, and PSI‐TKA Welch's *t*‐test determined whether the mean accuracy significantly differed from manual KA (bold* indicates *p* ≤ 0.05, not significant denoted by *italics*).

Study ID	*N*	Four femoral resections combined accuracy ± SD (mm)
**KA‐TKA manual instrumentation**	**385**	**0.175** ± **0.350 (ref.)**
RAA‐TKA cadaver [61, 70]	45	0.583* ± **0.504** ^*^
RAA‐TKA clinical [55, 60, 65, 69, 71]	214	0.795* ± **0.729** ^*^
RAA‐TKA combined [55, 60, 61, 65, 69, 70, 71]	259	0.754* ± **0.697** ^*^
PSI‐TKA combined [59, 64, 67, 68]	264	1.133* ± **1.171** ^*^

Abbreviations: KA, kinematic alignment; PSI, patient‐specific instrumentation; RAA, robotic arm‐assisted; TKA, total knee arthroplasty.

This literature review leads to the conclusion that PSI‐TKA is limited in its efficacy and may no longer have a role [1, 2, 3, 4, 5, 8, 12, 13, 18] and that RAA‐TKA may not be as accurate as we have been led to believe and deserves more rigorous study. Robotic users may be surprised that the present study and literature review demonstrate that a manual resection technique achieves greater accuracy than reported for CT‐based robotic technology. Analysing the robot's several potential error sources [8, 20, 21, 22, 23, 24, 25, 26, 27, 72, 73] can help elucidate this discrepancy. One source of error is introduced when creating 3D models from image segmentation. 3D bone models created from 0.625 mm and 1.25 mm CT slices average approximately 0.2 mm smaller than the patient's anatomy [21]. Other error sources are possible, though not explored in the literature, including the reproducibility of the pre‐surgical positioning of the femoral component on the 3D model, as well as variations and changes over time in the tolerances of the mechanical joints of the robotic arm. Sources of error may also arise during registration and verification [8, 20, 24, 72], which depend on repeatable anatomic registration as well as stable pin fixation and optical marker position throughout the procedure.

In comparison, the femoral resection guides are securely attached directly to the femur when manual instruments are employed for KA‐TKA, eliminating errors inherent to the robotic process, including those related to image acquisition, 3D model construction, component position planning, mechanical tolerances of all articulations, and patient‐to‐robot registration. Additionally, the cutting blocks and guides used in the present study have a removable saw captures with a larger interface, allowing for maximum pre‐cut visualisation and manual surgeon control. Furthermore, manual KA‐TKA, which focuses exclusively on the anatomic landmarks within the knee joint, avoids errors inherent in intramedullary or extramedullary guides targeting long‐axis limb alignment [8, 20, 74].

Howell compared the femoral resection accuracy of more and less experienced surgeons performing unrestricted caliper‐verified manual KA to PSI‐ and RAA‐TKA techniques, demonstrating that surgeons with less experience were statistically equivalent or more accurate compared to PSI‐ and RAA‐TKA, and that surgeons who had performed more than fifty caliper‐verified KA surgeries were significantly more accurate [54]. Thus, the femoral resection accuracy of caliper‐verified KA with manual instrumentation does not depend on surgeon experience to match or exceed the accuracy of PSI‐ and RAA‐TKA. Another report corroborates a minimal learning curve to obtain resection accuracy and reveals that operative time drops substantially over the course of the first thirty cases [52].

One weakness of the present study is that the analysis is limited to femoral resections. The tibial resection is also an essential element. We focused on the femur as the three‐dimensional shape of the tibial plateau creates measurement challenges, and there is a lack of studies with similar methodology in the literature. Analysis of tibial resections may require a different measurement methodology, possibly with three‐dimensional imaging and/or computer‐assisted algorithms.

Another limitation of the present study is the lack of clinical outcomes; however, the scope of this study was focused narrowly on the technical performance of the femoral resections, evaluating their accuracy and precision, utilising the manual tools and techniques described in the Methods, and contrasting our results with all comparable data in the literature. Clinical outcomes are outside of the scope of this manuscript and are not present in the comparable studies, though warrant future study.

Our findings evaluate resection data produced from a specific surgical technique (unrestricted caliper‐verified KA) and thus may not be directly comparable to data obtained with other techniques such as MA. KA and MA aim for different overall results and have different bone resection targets [31]. KA attempts to restore individual patients' natural kinematic axes, while MA seeks to achieve a neutral mechanical axis and a perpendicular joint line. We avoid directly comparing these alignment philosophies by focusing solely on the accuracy and precision of the technical performance of the femoral resections. Bias is eliminated by limiting the analysis to target versus actual resected measurements, irrespective of differing alignment philosophies and surgical techniques.

Despite disparate alignment goals or surgical techniques, each resection must be accurate and repeatable. Resections performed with a saw blade‐controlled manually or by a robotic arm‐will inevitably have some inherent error. There must be some tolerance built into the saw capture, such that the dimension of the capture is slightly larger than the saw blade, which will cause some variability, regardless of whether a human or robot is controlling the saw blade. Additionally, in the case of a saw blade operated by a robotic arm, there is also inherent variability or “slop” built into the system [8, 20, 21, 22, 23, 24, 25, 26, 27, 50, 72, 73, 75].

Regardless of technique, the threshold for recutting an undercut resection or supplementing an overcut resection with a washer or shift may affect accuracy. We used a recut threshold of ≥ 1.0 mm in the present study. Other surgeons using this KA technique may conduct recuts for different target versus actual difference thresholds, potentially resulting in variations in accuracy and precision.

Most PSI‐ and RAA‐TKA studies do not include resection thickness measurements; instead, they limit their reported data to the accuracy of angular targets, with that target generally being the achievement of a neutral HKA. Therefore, the accuracy of manual KA procedures in terms of matching an alignment target should be evaluated and compared to the RAA‐TKA literature, but this is outside of the scope of the present manuscript, and would require the calculation of a target postoperative HKA as well as target joint line obliquity.

One could argue that the outcomes of manually instrumented, caliper‐verified KA do not support its widespread adoption; however, the literature is replete with reports demonstrating excellent mid‐ to long‐term results [2, 76, 77, 78, 79, 80, 81, 82, 83, 84, 85, 86, 87, 88, 89, 90, 91]. In contrast, the most defensible conclusions supported by the knee robotics literature are that alignment outliers and iatrogenic soft tissue injury may be reduced, and possibly, very short‐term outcomes, such as length of stay, improved. Most notably, no blinded, randomised trials or comparative cohort trials demonstrate clinically meaningful improvements in mid‐ to long‐term clinical outcomes or implant survivorship, and much literature concludes the contrary [6, 7, 9, 10, 11, 14, 15, 16, 17, 19, 92, 93, 94, 95, 96, 97].

Although evidence suggests a relationship between the accuracy of bone resections and patient outcomes, the degree of accuracy required for positive outcomes remains unknown. There is no consensus regarding the best alignment target, and until that is conclusively determined, the evaluation of accuracy as the primary focus is misguided, without being sure of the best target. The rapid adoption of technology assistance in knee arthroplasty, primarily RAA‐TKA, has been driven mainly by non‐scientific media promotion and consequent consumer demand [6, 98]. Future studies must explore whether differences in bone resection accuracy translate to clinically meaningful differences in outcomes, including assessing the point of diminishing returns, where increased femoral resection accuracy may no longer yield benefits in outcomes.

## CONCLUSION

Caliper‐verified kinematic alignment TKA performed with manual instrumentation produces excellent femoral resection accuracy and precision, with the absolute difference between target and actual resections averaging less than 0.175 mm. These results match or exceed the resection accuracy of PSI‐ and RAA‐TKA, questioning the necessity of these costlier technologies. Future randomised trials are needed to clarify the impact of TKA femoral resection accuracy on long‐term outcomes, and whether technology‐assistance is beneficial.

## AUTHOR CONTRIBUTIONS

David F. Scott designed the study and performed writing—original draft and review. Emma N. Horton participated in review and performed data collection and analysis. Both authors read and approved the final manuscript.

## CONFLICT OF INTEREST STATEMENT

The corresponding author receives research support from Medacta and MicroPort.

## ETHICS STATEMENT

This study was approved by the Institutional Review Board of WCG IRB, study approval # 20200306. All patients signed informed consent.

## Data Availability

The data presented in this study may be available upon request to the corresponding author. The data are not publicly available due to privacy and ethical reasons.

## References

[1] Bardakos N , Lilikakis A . Customised jigs in primary total knee replacement. Orthopedic Muscul Sys Curr Res. 2014;1(S1):9007.

[2] Calliess T , Bauer K , Stukenborg‐Colsman C , Windhagen H , Budde S , Ettinger M . PSI kinematic versus non‐PSI mechanical alignment in total knee arthroplasty: a prospective, randomized study. Knee Surg Sports Traumatol Arthrosc. 2017;25(6):1743–1748. 27120192 10.1007/s00167-016-4136-8

[3] Chen JY , Yeo SJ , Yew AKS , Tay DKJ , Chia SL , Lo NN , et al. The radiological outcomes of patient‐specific instrumentation versus conventional total knee arthroplasty. Knee Surg Sports Traumatol Arthrosc. 2014;22(3):630–635. 23996069 10.1007/s00167-013-2638-1

[4] Conteduca F , Iorio R , Mazza D , Caperna L , Bolle G , Argento G , et al. Are MRI‐based, patient matched cutting jigs as accurate as the tibial guides? Int Orthop. 2012;36(8):1589–1593. 22426932 10.1007/s00264-012-1522-9 PMC3535040

[5] Conteduca F , Iorio R , Mazza D , Caperna L , Bolle G , Argento G , et al. Evaluation of the accuracy of a patient‐specific instrumentation by navigation. Knee Surg Sports Traumatol Arthrosc. 2013;21(10):2194–2199. 22735977 10.1007/s00167-012-2098-z

[6] Ekhtiari S , Sun B , Sidhu R , Ade‐Conde AM , Chaudhry H , Tomescu S , et al. Evidence versus frenzy in robotic total knee arthroplasty: a systematic review comparing news media claims to randomized controlled trial evidence. J Bone Jt Surg. 2024;106(24):2384–2392. 10.2106/JBJS.24.00264 39692716

[7] Fozo ZA , Ghazal AH , Hesham Gamal M , Matar SG , Kamal I , Ragab KM . A systematic review and meta‐analysis of conventional versus robotic‐assisted total knee arthroplasty. Cureus. 2023;15(10):e46845. 37869051 10.7759/cureus.46845 PMC10589058

[8] Hazratwala K , Brereton SG , Grant A , Dlaska CE . Computer‐assisted technologies in arthroplasty: navigating your way today. JBJS Rev. 2020;8(3):e0157. 32224641 10.2106/JBJS.RVW.19.00157

[9] Jeon SW , Kim KI , Song SJ . Robot‐assisted total knee arthroplasty does not improve long‐term clinical and radiologic outcomes. J Arthroplasty. 2019;34(8):1656–1661. 31036450 10.1016/j.arth.2019.04.007

[10] Kayani B , Konan S , Ayuob A , Onochie E , Al‐Jabri T , Haddad FS . Robotic technology in total knee arthroplasty: a systematic review. EFORT Open Rev. 2019;4(10):611–617. 31754467 10.1302/2058-5241.4.190022 PMC6836078

[11] Kirchner GJ , Stambough JB , Jimenez E , Mullen K , Nikkel LE . Robotic assistance is not associated with decreased early revisions in cementless TKA: an analysis of the American Joint Replacement Registry. Clin Orthop Relat Res. 2024;482(2):303–310. 39569799 10.1097/CORR.0000000000003330 PMC11828033

[12] Klatt BA , Goyal N , Austin MS , Hozack WJ . Custom‐fit total knee arthroplasty (OtisKnee) results in malalignment. J Arthroplasty. 2008;23(1):26–29. 18165024 10.1016/j.arth.2007.10.001

[13] Lustig S , Scholes CJ , Oussedik SI , Kinzel V , Coolican MRJ , Parker DA . Unsatisfactory accuracy as determined by computer navigation of VISIONAIRE patient‐specific instrumentation for total knee arthroplasty. J Arthroplasty. 2013;28(3):469–473. 23151366 10.1016/j.arth.2012.07.012

[14] Nogalo C , Meena A , Abermann E , Fink C . Complications and downsides of the robotic total knee arthroplasty: a systematic review. Knee Surg Sports Traumatol Arthrosc. 2023;31(3):736–750. 35716186 10.1007/s00167-022-07031-1 PMC9958158

[15] Sherman WF , Wu VJ . Robotic surgery in total joint arthroplasty: a survey of the AAHKS membership to understand the utilization, motivations, and perceptions of total joint surgeons. J Arthroplasty. 2020;35(12):3474–3481.e2. 32731999 10.1016/j.arth.2020.06.072

[16] Siddiqi A , Horan T , Molloy RM , Bloomfield MR , Patel PD , Piuzzi NS . A clinical review of robotic navigation in total knee arthroplasty: historical systems to modern design. EFORT Open Rev. 2021;6(4):252–269. 34040803 10.1302/2058-5241.6.200071 PMC8142596

[17] Tompkins GS , Sypher KS , Li HF , Griffin TM , Duwelius PJ . Robotic versus manual total knee arthroplasty in high volume surgeons: a comparison of cost and quality metrics. J Arthroplasty. 2022;37(8s):S782–S789. 34952162 10.1016/j.arth.2021.12.018

[18] Victor J , Dujardin J , Vandenneucker H , Arnout N , Bellemans J . Patient‐specific guides do not improve accuracy in total knee arthroplasty: a prospective randomized controlled trial. Clin Orthop Relat Res. 2014;472(1):263–271. 23616267 10.1007/s11999-013-2997-4 PMC3889461

[19] Yoon TJ , Kim TT . The role of advertising in high‐tech medical procedures: evidence from robotic surgeries. J Mark. 2024;88(1):97–115.

[20] Biant LC , Yeoh K , Walker PM , Bruce WJM , Walsh WR . The accuracy of bone resections made during computer navigated total knee replacement. Do we resect what the computer plans we resect? Knee. 2008;15(3):238–241. 18358725 10.1016/j.knee.2008.01.012

[21] Campanelli V , Howell SM , Hull ML . Morphological errors in 3D bone models of the distal femur and proximal tibia generated from magnetic resonance imaging and computed tomography determined using two registration methods. Comp Methods Biomech Biomed Eng: Imag Vis. 2020;8(1):31.

[22] Fontalis A , Hansjee S , Giebaly DE , Mancino F , Plastow R , Haddad FS . Troubleshooting robotics during total hip and knee arthroplasty. Orthop Clin North Am. 2024;55(1):33–48. 37980102 10.1016/j.ocl.2023.06.004

[23] Kim TK , Chang CB , Kang YG , Chung BJ , Cho HJ , Seong SC . Execution accuracy of bone resection and implant fixation in computer assisted minimally invasive total knee arthroplasty. Knee. 2010;17(1):23–28. 19581096 10.1016/j.knee.2009.06.004

[24] Lustig S , Fleury C , Goy D , Neyret P , Donell ST . The accuracy of acquisition of an imageless computer‐assisted system and its implication for knee arthroplasty. Knee. 2011;18(1):15–20. 20060724 10.1016/j.knee.2009.12.010

[25] Oberst M , Bertsch C , Lahm A , Wuerstlin S , Holz U . Regression and correlation analysis of preoperative versus intraoperative assessment of axes during navigated total knee arthroplasty. Comput Aided Surg. 2006;11(2):87–91. 16782644 10.3109/10929080600632680

[26] Singh V , Teo GM , Long WJ . Versatility and accuracy of a novel image‐free robotic‐assisted system for total knee arthroplasty. Arch Orthop Trauma Surg. 2021;141(12):2077–2086. 34255174 10.1007/s00402-021-04049-x

[27] Sires JD , Wilson CJ . CT Validation of Intraoperative Implant Position and Knee Alignment as Determined by the MAKO Total Knee Arthroplasty System. J Knee Surg. 2021;34(10):1133–1137. 32131103 10.1055/s-0040-1701447

[28] Howell SM , Hull ML , Nedopil AJ , Rivière C . Caliper‐verified kinematically aligned total knee arthroplasty: rationale, targets, accuracy, balancing, implant survival, and outcomes. Instr Course Lect. 2023;72:241–259. 36534860

[29] Howell SM , Papadopoulos S , Kuznik KT , Hull ML . Accurate alignment and high function after kinematically aligned TKA performed with generic instruments. Knee Surg Sports Traumatol Arthrosc. 2013;21(10):2271–2280. 23948721 10.1007/s00167-013-2621-x

[30] Howell SMHM . Principles of kinematic alignment in total knee arthroplasty with and without patient specific cutting blocks (OtisKnee). Insall and Scott Surgery of the Knee. 5th ed. 1. Philadelphia: Elsevier; 2012. p. 1255.

[31] Rivière C , Iranpour F , Auvinet E , Howell S , Vendittoli PA , Cobb J , et al. Alignment options for total knee arthroplasty: A systematic review. Orthopaedics & Traumatology, Surgery & Research: OTSR. 2017;103(7):1047–1056. 10.1016/j.otsr.2017.07.010 28864235

[32] Scott DF , Howell SM . Kinematic Alignment Possible With Manual Instrumentation, Medially Stabilized Implants. *Orthopedics Today*. 2019. https://www.healio.com/news/orthopedics/0211/kinematic

[33] Kayani B , Konan S , Huq SS , Tahmassebi J , Haddad FS . Robotic‐arm assisted total knee arthroplasty has a learning curve of seven cases for integration into the surgical workflow but no learning curve effect for accuracy of implant positioning. Knee Surg Sports Traumatol Arthrosc. 2019;27(4):1132–1141. 30225554 10.1007/s00167-018-5138-5 PMC6435632

[34] Christen B , Tanner L , Ettinger M , Bonnin MP , Koch PP , Calliess T . Comparative cost analysis of four different computer‐assisted technologies to implant a total knee arthroplasty over conventional instrumentation. J Pers Med. 2022;12(2):184. 35207672 10.3390/jpm12020184 PMC8880057

[35] Ezeokoli EU , John J , Gupta R , Jawad A , Cavinatto L . Index surgery and ninety day re‐operation cost comparison of robotic‐assisted versus manual total knee arthroplasty. Int Orthop. 2023;47(2):359–364. 36574020 10.1007/s00264-022-05674-w

[36] Kolessar D , Hayes D , Harding J , Rudraraju R , Graham J . Robotic‐arm assisted technology's impact on knee arthroplasty and associated healthcare costs. J Health Econ Outcomes Res. 2022;9(2):57–66. 36072348 10.36469/001c.37024 PMC9398468

[37] Moschetti WE , Konopka JF , Rubash HE , Genuario JW . Can robot‐assisted unicompartmental knee arthroplasty be cost‐effective? A Markov decision analysis. J Arthroplasty. 2016;31(4):759–765. 26706836 10.1016/j.arth.2015.10.018

[38] Steffens D , Karunaratne S , McBride K , Gupta S , Horsley M , Fritsch B . Implementation of robotic‐assisted total knee arthroplasty in the public health system: a comparative cost analysis. Int Orthop. 2022;46(3):481–488. 34549322 10.1007/s00264-021-05203-1

[39] Tompkins GS , Sypher KS , Griffin TM , Duwelius PD . Can a reduction in revision rates make robotic total knee arthroplasty cost neutral with manual total knee arthroplasty at ten‐year follow‐up? An episode cost analysis. J Arthroplasty. 2022;37(8s):S777–S781.e3. 34808279 10.1016/j.arth.2021.10.030

[40] Bollars P , Boeckxstaens A , Mievis J , Kalaai S , Schotanus MGM , Janssen D . Preliminary experience with an image‐free handheld robot for total knee arthroplasty: 77 cases compared with a matched control group. Eur J Orthop Surg Traumatol. 2020;30(4):723–729. 31950265 10.1007/s00590-020-02624-3

[41] Bollars P , Janssen D , De Weerdt W , Albelooshi A , Meshram P , Nguyen TD , et al. Improved accuracy of implant placement with an imageless handheld robotic system compared to conventional instrumentation in patients undergoing total knee arthroplasty: a prospective randomized controlled trial using CT‐based assessment of radiological outcomes. Knee Surgery Sports Traumatol Arthrosc. 2023;31(12):5446–5452. 10.1007/s00167-023-07590-x 37796307

[42] Cheng R , Kim B , Taylor WL , Westrich GH , Shen TS . Robotic‐assisted total knee arthroplasty is associated with the use of thinner polyethylene liners compared to navigation‐guided and manual techniques. Knee Surg Sports Traumatol Arthrosc. 2024;32(9):2290–2296. 38738862 10.1002/ksa.12228

[43] Clement ND , Galloway S , Baron YJ , Smith K , Weir DJ , Deehan DJ . Robotic arm‐assisted versus manual (ROAM) total knee arthroplasty: a randomized controlled trial. Bone Joint J. 2023;105–b(9):961–970. 10.1302/0301-620X.105B9.BJJ-2023-0006.R3 37652449

[44] Deckey DG , Rosenow CS , Verhey JT , Brinkman JC , Mayfield CK , Clarke HD , et al. Robotic‐assisted total knee arthroplasty improves accuracy and precision compared to conventional techniques. Bone Joint J. 2021;103–b(6 Supple A):74–80. 10.1302/0301-620X.103B6.BJJ-2020-2003.R1 34053292

[45] Mahoney O , Kinsey T , Sodhi N , Mont MA , Chen AF , Orozco F , et al. Improved component placement accuracy with robotic‐arm assisted total knee arthroplasty. J Knee Surg. 2022;35(03):337–344. 32869232 10.1055/s-0040-1715571

[46] Turan K , Camurcu Y , Kezer M , Uysal Y , Kizilay YO , Ucpunar H , et al. A comparison of robotic‐assisted and manual techniques in restricted kinematically aligned total knee arthroplasty: coronal alignment improvement with no significant clinical differences. Knee Surg Sports Traumatol Arthrosc. 2023;31(11):4673–4679. 37165209 10.1007/s00167-023-07426-8

[47] Goyal N , Patel AR , Yaffe MA , Luo MY , Stulberg SD . Does implant design influence the accuracy of patient specific instrumentation in total knee arthroplasty? J Arthroplasty. 2015;30(9):1526–1530. 25861920 10.1016/j.arth.2015.03.019

[48] Mattei L , Pellegrino P , Calò M , Bistolfi A , Castoldi F . Patient specific instrumentation in total knee arthroplasty: a state of the art. Ann Transl Med. 2016;4(7):126. 27162776 10.21037/atm.2016.03.33 PMC4842392

[49] Török L , Jávor P , Hartmann P , Bánki L , Varga E . Should we abandon the patient‐specific instrumentation ship in total knee arthroplasty? Not quite yet! BMC Musculoskelet Disord. 2021;22(1):730. 34429099 10.1186/s12891-021-04581-2 PMC8386088

[50] Klasan A , Putnis SE , Grasso S , Neri T , Coolican MR . Conventional instruments are more accurate for measuring the depth of the tibial cut than computer‐assisted surgery in total knee arthroplasty: a prospective study. Arch Orthop Trauma Surg. 2020;140(6):801–806. 32146591 10.1007/s00402-020-03403-9

[51] Levy YD , An VVG , Shean CJW , Groen FR , Walker PM , Bruce WJM . The accuracy of bony resection from patient‐specific guides during total knee arthroplasty. Knee Surg Sports Traumatol Arthrosc. 2017;25(6):1678–1685. 27492384 10.1007/s00167-016-4254-3

[52] Nedopil AJ , Dhaliwal A , Howell SM , Hull ML . A surgeon that switched to unrestricted kinematic alignment with manual instruments has a short learning curve and comparable resection accuracy and outcomes to those of an experienced surgeon. J Pers Med. 2022;12(7):1152. 35887649 10.3390/jpm12071152 PMC9320158

[53] Nizam I , Batra AV . Accuracy of bone resection in total knee arthroplasty using CT assisted‐3D printed patient specific cutting guides. SICOT‐J. 2018;4:29. 30009760 10.1051/sicotj/2018032 PMC6047362

[54] Howell SM , Nedopil AJ , Hull ML . Negligible effect of surgeon experience on the accuracy and time to perform unrestricted caliper verified kinematically aligned TKA with manual instruments. Knee Surg Sports Traumatol Arthrosc. 2022;30:2966–2974. 35366075 10.1007/s00167-022-06939-y PMC9418297

[55] Li C , Zhang Z , Wang G , Rong C , Zhu W , Lu X , et al. Accuracies of bone resection, implant position, and limb alignment in robotic‐arm‐assisted total knee arthroplasty: a prospective single‐centre study. J Orthop Surg. 2022;17(1):61. 10.1186/s13018-022-02957-1 PMC8800350 35093133

[56] Parratte S , Price AJ , Jeys LM , Jackson WF , Clarke HD . Accuracy of a new robotically assisted technique for total knee arthroplasty: a cadaveric study. J Arthroplasty. 2019;34(11):2799–2803. 31301912 10.1016/j.arth.2019.06.040

[57] Lee HJ , Park KK , Park YB , Choi SW , Kim BO , Kim SH . Accuracy of advanced active robot for total knee arthroplasty: a cadaveric study. J Knee Surg. 2024;37(2):135–141. 36638805 10.1055/s-0042-1760391

[58] Sires JD , Craik JD , Wilson CJ . Accuracy of bone resection in MAKO total knee robotic‐assisted surgery. J Knee Surg. 2021;34(7):745–748. 31694057 10.1055/s-0039-1700570

[59] Kim KK , Song J . Accuracy of patient‐specific instrument for cylindrical axis implementation in kinematically aligned total knee arthroplasty. Clin Orthop Surg. 2023;15(5):760. 37811500 10.4055/cios22147 PMC10551691

[60] Gamie Z , Kenanidis E , Douvlis G , Milonakis N , Maslaris A , Tsiridis E . Accuracy of the imageless mode of the ROSA robotic system for targeted resection thickness in total knee arthroplasty: a prospective, single surgeon case‐series study. Int J Med Robot Comput Assist Surg. 2024;20(6):e70029. 10.1002/rcs.70029 PMC11666924 39716397

[61] Seidenstein A , Birmingham M , Foran J , Ogden S . Better accuracy and reproducibility of a new robotically‐assisted system for total knee arthroplasty compared to conventional instrumentation: a cadaveric study. Knee Surg Sports Traumatol Arthrosc. 2021;29(3):859–866. 32448945 10.1007/s00167-020-06038-w

[62] Adkar N , Patil M , Vaidya S , Kumbar R , Kerhalkar R , Mote G , et al. Correlation between planned and executed bone cuts using robotics in total knee arthroplasty: a prospective study of 500 patients. Indian J Orthop. 2024;58(8):1103–1108. 39087031 10.1007/s43465-024-01196-2 PMC11286900

[63] Yi J , Gao Z , Huang Y , Liu Y , Zhang Y , Chai W . Evaluating the accuracy of a new robotically assisted system in cadaveric total knee arthroplasty procedures. J Orthop Surg. 2024;19(1):354. 10.1186/s13018-024-04788-8 PMC11179344 38879524

[64] Yamamura K , Inori F , Konishi S . Evaluation of the accuracy of resected bone thickness based on patient‐specific instrumentation during total knee arthroplasty. Arch Orthop Trauma Surg. 2021;141(9):1583–1590. 33547928 10.1007/s00402-021-03805-3

[65] Rossi SMP , Sangaletti R , Perticarini L , Terragnoli F , Benazzo F . High accuracy of a new robotically assisted technique for total knee arthroplasty: an in vivo study. Knee Surg Sports Traumatol Arthrosc. 2023;31(3):1153–1161. 34981162 10.1007/s00167-021-06800-8 PMC8723813

[66] Schrednitzki D , Horn CE , Lampe UA , Halder AM . Imageless robotic‐assisted total knee arthroplasty is accurate in vivo: a retrospective study to measure the postoperative bone resection and alignment. Arch Orthop Trauma Surg. 2023;143(6):3471–3479. 36269397 10.1007/s00402-022-04648-2

[67] Kang DG , Kim KI , Bae JK . MRI‐based or CT‐based patient‐specific instrumentation in Total knee Arthroplasty: How do the two systems compare? Arthroplasty. 2020;2(1):1. 35236432 10.1186/s42836-019-0020-6 PMC8796460

[68] Wernecke GC , Taylor S , Wernecke P , MacDessi SJ , Chen DB . Resection accuracy of patient‐specific cutting guides in total knee replacement. ANZ J Surg. 2017;87(11):921–924. 28853192 10.1111/ans.14143

[69] Wan X , Su Q , Wang D , Yuan M , Lai Y , Xu H , et al. Robotic arm‐assisted total knee arthroplasty improves preoperative planning and intraoperative decision‐making. J Orthop Surg. 2021;16(1):670. 10.1186/s13018-021-02815-6 PMC8591833 34781977

[70] Xia R , Tong Z , Hu Y , Kong K , Wu X , Li H . Skywalker’ surgical robot for total knee arthroplasty: An experimental sawbone study. Int J Med Robot Comput Assist Surg. 2021;17(5):e2292. 10.1002/rcs.2292 34058058

[71] Xia R , Zhai Z , Zhang J , Yu D , Wang L , Mao Y , et al. Verification and clinical translation of a newly designed “Skywalker” robot for total knee arthroplasty: a prospective clinical study. J Orthop Transl. 2021;29:143–151. 10.1016/j.jot.2021.05.006 PMC8242054 34249612

[72] Herregodts S , Vermue H , Herregodts J , De Coninck B , Chevalier A , Verstraete M , et al. Accuracy of intraoperative bone registration and stereotactic boundary reconstruction during total knee arthroplasty surgery. Int J Med Robot Comput Assist Surg. 2023;19(1):e2460. 10.1002/rcs.2460 36088533

[73] Martensson NE . Identifying sources of error in computer navigated total knee arthroplasties using a metric on Se(3) and sensitivity analyses. In: The University of Western Ontario. Canada: ProQuest Dissertations & Theses; 2023.

[74] Maestro A , Harwin SF , Sandoval MG , Vaquero DH , Murcia A . Influence of intramedullary versus extramedullary alignment guides on final total knee arthroplasty component position. J Arthroplasty. 1998;13(5):552–558. 9726321 10.1016/s0883-5403(98)90055-9

[75] Nishihara S , Sugano N , Ikai M , Sasama T , Tamura Y , Tamura S , et al. Accuracy evaluation of a shape‐based registration method for a computer navigation system for total knee arthroplasty. J Knee Surg. 2003;16(2):98–105. 12741423

[76] An VVG , Twiggs J , Leie M , Fritsch BA . Kinematic alignment is bone and soft tissue preserving compared to mechanical alignment in total knee arthroplasty. Knee. 2019;26(2):466–476. 30772187 10.1016/j.knee.2019.01.002

[77] Blakeney W , Clément J , Desmeules F , Hagemeister N , Rivière C , Vendittoli PA . Kinematic alignment in total knee arthroplasty better reproduces normal gait than mechanical alignment. Knee Surg Sports Traumatol Arthrosc. 2019;27(5):1410–1417. 30276435 10.1007/s00167-018-5174-1

[78] Dossett HG , Arthur JR , Makovicka JL , Mara KC , Bingham JS , Clarke HD , et al. A randomized controlled trial of kinematically and mechanically aligned total knee arthroplasties: long‐term follow‐up. J Arthroplasty. 2023;38(6S):S209–S214. 37003458 10.1016/j.arth.2023.03.065

[79] Dossett HG , Estrada NA , Swartz GJ , LeFevre GW , Kwasman BG . A randomised controlled trial of kinematically and mechanically aligned total knee replacements: two‐year clinical results. Bone Joint J. 2014;96–b(7):907–913. 10.1302/0301-620X.96B7.32812 24986944

[80] Elbuluk AM , Jerabek SA , Suhardi VJ , Sculco PK , Ast MP , Vigdorchik JM . Head‐to‐head comparison of kinematic alignment versus mechanical alignment for total knee arthroplasty. J Arthroplasty. 2022;37(8S):S849–S851. 35093548 10.1016/j.arth.2022.01.052

[81] Ettinger M , Tuecking LR , Savov P , Windhagen H . Higher satisfaction and function scores in restricted kinematic alignment versus mechanical alignment with medial pivot design total knee arthroplasty: A prospective randomised controlled trial. Knee Surg Sports Traumatol Arthrosc. 2024;32(5):1275–1286. 38501253 10.1002/ksa.12143

[82] Jeremić DV , Massouh WM , Sivaloganathan S , Rosali AR , Haaker RG , Rivière C . Short‐term follow‐up of kinematically vs. mechanically aligned total knee arthroplasty with medial pivot components: a case‐control study. Orthop Traumatol Surg Res. 2020;106(5):921–927. 32522532 10.1016/j.otsr.2020.04.005

[83] MacDessi SJ , Griffiths‐Jones W , Chen DB , Griffiths‐Jones S , Wood JA , Diwan AD , et al. Restoring the constitutional alignment with a restrictive kinematic protocol improves quantitative soft‐tissue balance in total knee arthroplasty: a randomized controlled trial. Bone Joint J. 2020;102–B(1):117–124. 10.1302/0301-620X.102B1.BJJ-2019-0674.R2 PMC6974544 31888372

[84] Matsumoto T , Takayama K , Ishida K , Hayashi S , Hashimoto S , Kuroda R . Radiological and clinical comparison of kinematically versus mechanically aligned total knee arthroplasty. Bone Joint J. 2017;99–b(5):640–646. 10.1302/0301-620X.99B5.BJJ-2016-0688.R2 28455473

[85] McEwen PJ , Dlaska CE , Jovanovic IA , Doma K , Brandon BJ . Computer‐assisted kinematic and mechanical axis total knee arthroplasty: a prospective randomized controlled trial of bilateral simultaneous surgery. J Arthroplasty. 2020;35(2):443–450. 31591010 10.1016/j.arth.2019.08.064

[86] Niki Y , Nagura T , Kobayashi S , Udagawa K , Harato K . Who will benefit from kinematically aligned total knee arthroplasty? perspectives on patient‐reported outcome measures. J Arthroplasty. 2020;35(2):438–442.e2. 31668528 10.1016/j.arth.2019.09.035

[87] Sarzaeem MM , Movahedinia M , Mirahmadi A , Abolghasemian M , Tavakoli M , Omrani A , et al. Kinematic alignment technique outperforms mechanical alignment in simultaneous bilateral total knee arthroplasty: a randomized controlled trial. J Arthroplasty. 2024;39(9):2234–2240. 38537837 10.1016/j.arth.2024.03.045

[88] Shelton TJ , Gill M , Athwal G , Howell SM , Hull ML . Outcomes in patients with a calipered kinematically aligned TKA that already had a contralateral mechanically aligned TKA. J Knee Surg. 2021;34(1):087–093. 10.1055/s-0039-1693000 31288274

[89] Wen L . An early clinical comparative study on total knee arthroplasty with kinematic alignment using specific instruments versus mechanical alignment in varus knees. Frontiers. 2023;9:1097302. 10.3389/fsurg.2022.1097302 PMC9889970 36743893

[90] Won SH , Eim SH , Shen QH , Kim KK , Won YY . Caliper‐verified unrestricted kinematically aligned total knee arthroplasty in Asian patients showed efficacious mid‐ to long‐term results regardless of postoperative alignment categories. Knee Surg Sports Traumatol Arthrosc. 2024;32(4):941–952. 38461403 10.1002/ksa.12117

[91] Bar Ziv Y , Small I , Keidan T , Beit Ner E , Agar G , Shohat N . Patients undergoing staged bilateral knee arthroplasty are less aware of their kinematic aligned knee compared to their mechanical knee. J Orthop. 2021;23:155–159. 33542593 10.1016/j.jor.2020.12.032 PMC7840796

[92] Agarwal N , To K , McDonnell S , Khan W . Clinical and radiological outcomes in robotic‐assisted total knee arthroplasty: a systematic review and meta‐analysis. J Arthroplasty. 2020;35(11):3393–3409.e2. 32234326 10.1016/j.arth.2020.03.005

[93] Batailler C , Fernandez A , Swan J , Servien E , Haddad FS , Catani F , et al. MAKO CT‐based robotic arm‐assisted system is a reliable procedure for total knee arthroplasty: a systematic review. Knee Surg Sports Traumatol Arthrosc. 2021;29(11):3585–3598. 32975626 10.1007/s00167-020-06283-z

[94] Bensa A , Sangiorgio A , Deabate L , Illuminati A , Pompa B , Filardo G . Robotic‐assisted mechanically aligned total knee arthroplasty does not lead to better clinical and radiological outcomes when compared to conventional TKA: a systematic review and meta‐analysis of randomized controlled trials. Knee Surg Sports Traumatol Arthrosc. 2023;31(11):4680–4691. 37270464 10.1007/s00167-023-07458-0

[95] Daoub A , Qayum K , Patel R , Selim A , Banerjee R . Robotic assisted versus conventional total knee arthroplasty: a systematic review and meta‐analysis of randomised controlled trials. J Robotic Surg. 2024;18(1):364. 10.1007/s11701-024-02048-9 39382767

[96] Fu X , She Y , Jin G , Liu C , Liu Z , Li W , et al. Comparison of robotic‐assisted total knee arthroplasty: an updated systematic review and meta‐analysis. J Robot Surg. 2024;18(1):292. 39052153 10.1007/s11701-024-02045-y PMC11272701

[97] Ren Y , Cao S , Wu J , Weng X , Feng B . Efficacy and reliability of active robotic‐assisted total knee arthroplasty compared with conventional total knee arthroplasty: a systematic review and meta‐analysis. Postgrad Med J. 2019;95(1121):125–133. 30808721 10.1136/postgradmedj-2018-136190 PMC6585281

[98] Brinkman JC , Christopher ZK , Moore ML , Pollock JR , Haglin JM , Bingham JS . Patient interest in robotic total joint arthroplasty is exponential: a 10‐year Google trends analysis. Arthroplast Today. 2022;15:13–18. 35360676 10.1016/j.artd.2022.02.015 PMC8961076

